# Airway management during missions of a neurologist-staffed mobile stroke unit: a retrospective registry study

**DOI:** 10.1186/s13049-026-01726-x

**Published:** 2026-11-09

**Authors:** Kian Röhrs, Heinrich J. Audebert, Joachim E. Weber

**Affiliations:** 1 Department of Neurology, Charité—Universitätsmedizin Berlin, Corporate Member of Freie Universität Berlin and Humboldt-Universität zu Berlin, Charitéplatz 1, 10117 Berlin, Germany; 2 Center for Stroke Research Berlin, Charité—Universitätsmedizin Berlin, Corporate Member of Freie Universität Berlin and Humboldt-Universität zu Berlin, Berlin, Germany; 3 Berlin Institute of Health at Charité—Universitätsmedizin Berlin, Berlin, Germany; 4 German Center for Cardiovascular Research (DZHK), Partner Site Berlin, Berlin, Germany

**Keywords:** Mobile stroke unit, Prehospital care, Airway management, Endotracheal intubation, Neurology, Emergency medical services, First-pass success, Workload

## Abstract

**Background:**

Following their endorsement by international guidelines, mobile stroke units (MSUs) are increasingly being integrated into emergency medical services worldwide. In real-world use, physician-staffed MSUs can be dispatched beyond their intended indication, exposing on-board neurologists to non-stroke emergencies. This raises important clinical and regulatory questions about the competencies MSU physicians need. Advanced airway management is one of the most critical prehospital interventions. Yet, its frequency, indications, procedural characteristics and success rates have not been investigated in neurologist-staffed MSU services.

**Methods:**

We retrospectively analysed a prospective registry of mission documentation from a neurologist-staffed MSU service in Berlin, Germany from 31 August 2024 to 15 March 2026. Dispatches of the MSU were classified as stroke or non-stroke dispatches using the recorded activation code. The primary outcome was the dispatch-based frequency of endotracheal intubation (ETI), defined as a documented intubation attempt. All ETIs were performed by the on-board neurologist. Secondary outcomes included supraglottic airway (SGA) use, first-pass success rate, procedural characteristics and self-reported post-mission National Aeronautics and Space Administration Task Load Index (NASA-TLX) workload. Proportions are reported with 95% Wilson confidence intervals (CI).

**Results:**

We analysed 3,887 dispatches. ETI occurred in a total of 49 dispatches (1.26%; 95% CI 0.95–1.66) with 2 in 1,755 stroke- (0.11%; 95% CI 0.03–0.41) and 47 in 2,132 non-stroke dispatches (2.20%; 95% CI 1.66–2.92). SGA use occurred in 22 dispatches (0.57%; 95% CI 0.37–0.86). All 49 intubation attempts were successful (100.0%; 95% CI 92.7–100.0). First-pass success was achieved in 43/49 (87.8%; 95% CI 75.8–94.3) with no ETI requiring more than two attempts. Videolaryngoscopy was used in all ETIs and capnographic confirmation of tube position in 48/49 (98%). Non-traumatic cardiac arrest was the most frequent indication (30/49). In the exploratory workload analysis, the median NASA-TLX score was 59.8 [IQR 50.0–68.7] in 49 dispatches with ETI and 27.0 [15.5–41.7] in 2,700 dispatches without ETI.

**Conclusions:**

ETI was infrequent overall and particularly rare during stroke dispatches, yet first-pass success was high. Prospective studies are needed to define airway management requirements and training standards for MSU physicians.

**Supplementary Information:**

The online version contains supplementary material available at https://doi.org/10.1186/s13049-026-01726-x.

## Background

Stroke accounts for a substantial proportion of the global neurological disease burden [1, 2]. Acute ischaemic stroke represents one of the most time-critical neurological emergencies [3, 4] as the therapeutic efficacy of reperfusion therapy declines almost exponentially within the first 60 min after symptom onset [5, 6]. This therapeutic imperative has driven the development of specialised ambulances called mobile stroke units (MSU), which were first conceptualised in 2003 [7]. MSUs combine specially trained personnel with a mobile CT-scanner, point-of-care laboratory testing and often times telemedicine to enable prehospital thrombolysis and targeted transfer to the appropriate level of care [8–10]. MSU treatment improves key treatment metrics, including shorter onset-to-treatment times and higher rates of thrombolysis [11], and two large prospective controlled trials demonstrated improved functional outcomes with MSU care ([12, 13]), which led to an endorsement by the European Stroke Organisation [14] and by the American Heart Association/American Stroke Association (AHA/ASA) guidelines [15]. MSUs are therefore increasingly being integrated into emergency medical services (EMS) worldwide, with 42 active programmes currently listed by the Prehospital Stroke Treatment Organization (PRESTO) [16].

Staffing models for MSUs vary between and often even within countries, with neurological expertise delivered either by telemedicine or by an on-board vascular neurologist with additional emergency medicine training [10, 17]. In systems using onboard physicians, this can result in real world dispatching practices outside of the originally intended use case by combining stroke dispatches with non-stroke dispatches [18, 19], for example to cardiac arrest, which represents another emergency to which the time-is-brain principle applies. The way an MSU service is organised and dispatched is likely to affect the case mix and how often teams perform critical care interventions for which they must be trained and prepared.

Advanced airway management like endotracheal intubation (ETI) requires particular attention in this setting. The procedure is invasive, technically demanding and directly affects patient safety. Operator experience and the limitations of working outside a structured hospital environment influence its performance [20]. In services in which MSUs are also dispatched to non-stroke emergencies, airway interventions are liekly to occur unequally across mission types. Despite a mature and growing literature on MSUs and on prehospital airway management, there is little specific evidence on airway management on MSUs and on airway management performed by neurologists in the prehospital setting. It currently is unknown how often ETI is performed in MSU practice, the clinical and operational circumstances in which it is necessary or the first-pass success rates usually achieved in these scenarios. For MSUs staffed by on-board neurologists, these questions are highly relevant because airway interventions may influence training requirements, staffing models, quality assurance and service design.

We retrospectively analysed a prospective mission registry of a neurologist-staffed MSU service in Berlin, Germany. The primary aim was to analyse how often ETI occurred per dispatch, both overall and in stroke versus non-stroke dispatches as classified by the dispatch center. We also examined supraglottic airway use, procedural ETI characteristics including first-pass success and self-reported workload measured with the NASA-TLX. By examining both dispatch-level patterns and individual procedures, we characterized the system-level frequency of airway management and the procedural burden associated with its use in this service.

## Methods

### Ethics approval

The study protocol was approved by the Ethics Committee of Charité - Universitätsmedizin Berlin (Application number: EA2/193/25). The approval covered the retrospective analysis of routine registry data without individual patient consent. The study was conducted in accordance with the Declaration of Helsinki.

### Study design and setting

This retrospective study used the continuously updated digital mission registry of a neurologist-staffed MSU (Stroke-Emergency-Mobile, STEMO 3600, Stroke Emergency Mobile) in Berlin, Germany. The registry is maintained prospectively and the analysis covered 31 August 2024 to 15 March 2026. The MSU is part of Berlins emergency medical system (Berlin Fire Brigade) and operates daily between 07:00 and 23:00. The MSU is dispatched to suspected strokes as a dedicated MSU or to other emergencies as a general emergency physician response vehicle, depending on the availability of other EMS resources and proximity to the incident. While highly specialised prehospital stroke care is the intended use case, dispatch as a general emergency physician vehicle is a current real world practice, which is accepted only as an interim solution, as dispatching algorithms are under constant development. MSUs in Berlin are staffed with a paramedic, a radiology technician and a neurologist qualified in emergency medicine (Zusatzbezeichnung Notfallmedizin). This qualification is distinct from a specialist certification in emergency medicine and from Advanced Cardiovascular Life Support (ACLS) certification alone. The formal qualification requirements for emergency physicians in Berlin apply irrespective of their primary clinical specialty. These requirements and the additional training and refresher requirements of the study service are described in Supplementary Methods S1. A conventional ambulance is routinely dispatched alongside the MSU. All ETIs (both primary and secondary) were performed by the on-board neurologist, while equipment was usually prepared by the MSU paramedic or a crew member of the ambulance. Rescue options included an SGA, bag-mask ventilation and emergency cricothyrotomy equipment available on board.

### Data source and documentation

The data source of this study was the digital mission log of the STEMO 3600, which is maintained as a mandatory operational record. We used REDCap (Research Electronic Data Capture) to maintain this log. RedCap provides a secure, metadata-driven environment for structured electronic data capture and database management, with standardised documentation, controlled user access, traceable data entry and export of curated datasets for analysis. Immediately after each mission, the on-board physician entered the structured mission-level data using an internet-capable iPad. Entries covered dispatch and operational variables, the working diagnosis, transport and destination, prehospital procedures including advanced airway management, selected procedural characteristics and self-assessed post-mission workload (NASA Task Load Index) [21]. Airway-related documentation includes specific information on indication, device selection, laryngoscopy technique, glottic view and difficult airway indicators, number of attempts, administered medication, use of waveform capnography, confirmation of tube position and complications. Capnography was recorded both as a monitoring modality and as a method of confirming tube position. Direct visualization during videolaryngoscopy was also used to confirm tube position.

### Study population and mission classification

All registry entries recorded during the study period were screened for eligibility. Three records were excluded before final analysis due to missing dispatch information. No further exclusions were made and the final analysis set comprised 3,887 dispatches. Inclusion was not restricted to adults. Age was not systematically recorded for all dispatches.

Mission type was classified using dispatch documentation fields. Dispatches were categorised as stroke dispatches when the dispatch code indicated a “STEMO” (Stroke Emergency Mobile, MSU) mission. All other dispatches were categorised as non-stroke dispatches. All dispatches concerned the same vehicle, operating either in its MSU role or as a general emergency physician response vehicle (NEF; Notarzteinsatzfahrzeug).

### Outcomes and definitions

Reporting follows STROBE principles for observational studies where applicable [22]. The primary outcome was the dispatch-based frequency of ETI, defined as an intubation attempt. The number of intubation attempts were recorded as unsuccessful or one to five attempts. Secondary outcomes were dispatch-based frequency of supraglottic airway use, number of ETI attempts, first-pass success, procedural characteristics of ETI including method of confirmation of tube position, glottic view and difficult airway indicators, airway-related complications and post-mission self-reported overall workload (NASA-TLX) [21]. The NASA-TLX score (“overall workload”) was calculated as the unweighted mean of the six domain ratings (mental demand, physical demand, temporal demand, performance, effort and frustration), resulting in a score from 0 to 100. Higher scores indicated greater perceived workload. The workload analysis included all recorded overall scores, 18 of which were based on fewer than six domain ratings.

SGA use was defined as any recorded use of a laryngeal tube, laryngeal mask, or i-gel^®^ second-generation supraglottic airway. An intubation attempt was defined as attempted laryngoscopy with the intent to intubate. More specifically the insertion of the laryngoscope blade into the mouth was defined as an attempt. The complete withdrawal followed by reinsertion was defined as a new attempt [23]. Overall intubation success was defined as placement of the endotracheal tube in the trachea at any attempt and first-pass success was defined as placement at the first attempt. Attempt-level data were only recorded for ETI and not for SGA use. Indications, airway devices, glottic view and difficult-airway indicators were documented as multiple-selection variables. Because categories were not mutually exclusive, category counts could exceed the total number of cases in certain analyses.

### Statistical analysis

All eligible dispatches during the study period were included. Data was exported from REDCap after data curation and plausibility checks. REDCap is an established electronic data capture platform for clinical research and registry-based data management [24, 25]. Statistical analyses were performed using GraphPad Prism version 11.0.0 (GraphPad Software, Boston, MA, USA).

Analyses were primarily descriptive. Categorical variables are reported as counts and percentages. Dispatch-based and procedure-based proportions are presented as n/N, with 95% Wilson confidence intervals for the primary and secondary frequency outcomes [26]. Continuous variables are summarised as median [interquartile range] unless otherwise stated. Exploratory group comparisons of continuous variables were performed using the Mann-Whitney U test. All tests were two-sided. P values were interpreted descriptively and no adjustment for multiple comparisons was performed. Patient characteristics and physician-level ETI frequencies were summarised descriptively, without formal subgroup testing. Physician-level ETI frequencies were calculated as ETI cases per 100 recorded dispatches attributed to each physician.

## Results

### Study cohort

**Fig. 1:**
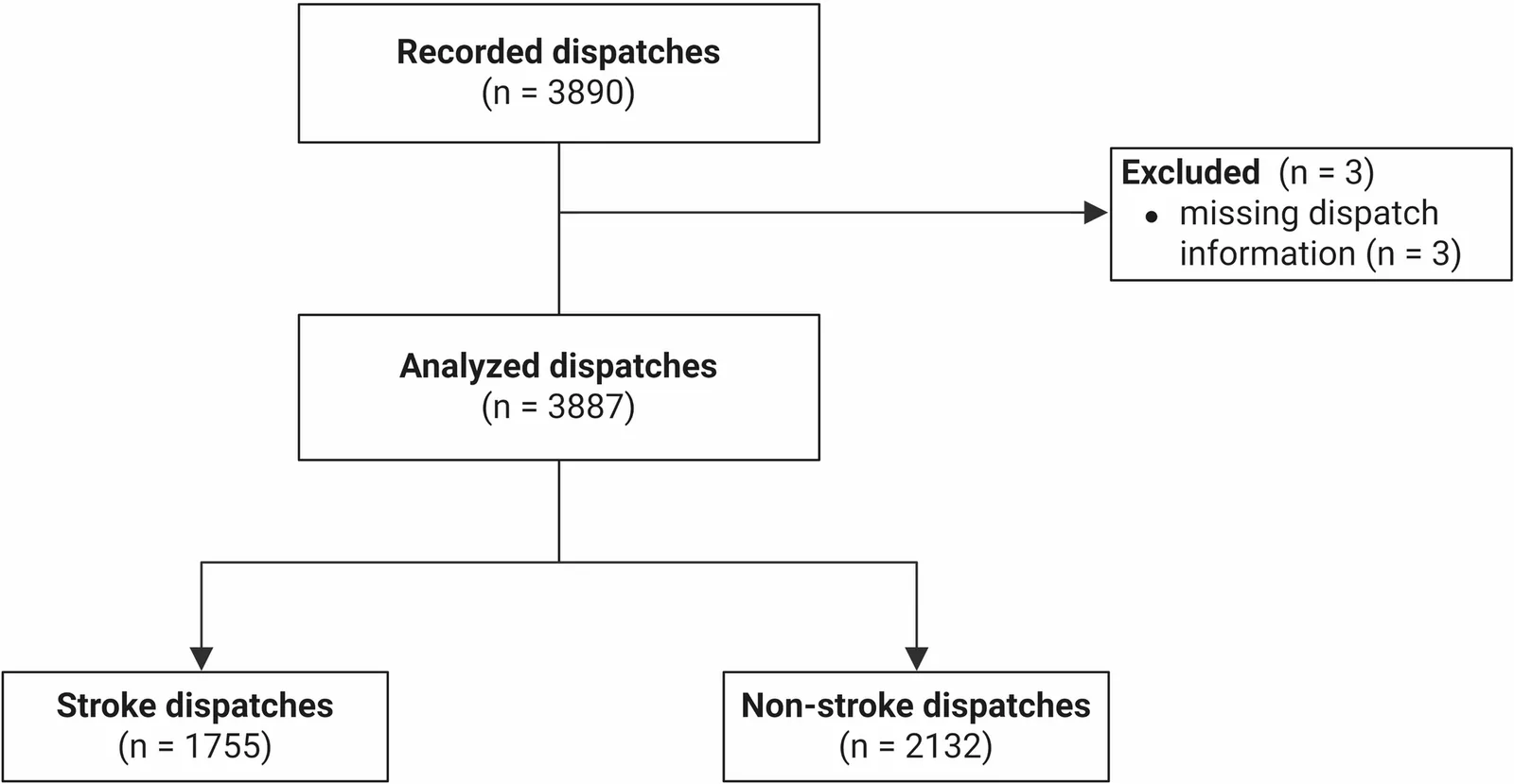
Study cohort and dispatch classification. Flow diagram of recorded dispatches, exclusions, and dispatch classification

The registry contained 3,890 recorded dispatches during the study period. Three records were excluded because dispatch information were missing. The final analysis included 3,887 dispatches (Fig. 1). Of these 1,755 (45.2%) were stroke dispatches and 2,132 (54.8%) were non-stroke dispatches.

### Dispatch-based frequency of ETI and SGA

**Fig. 2:**
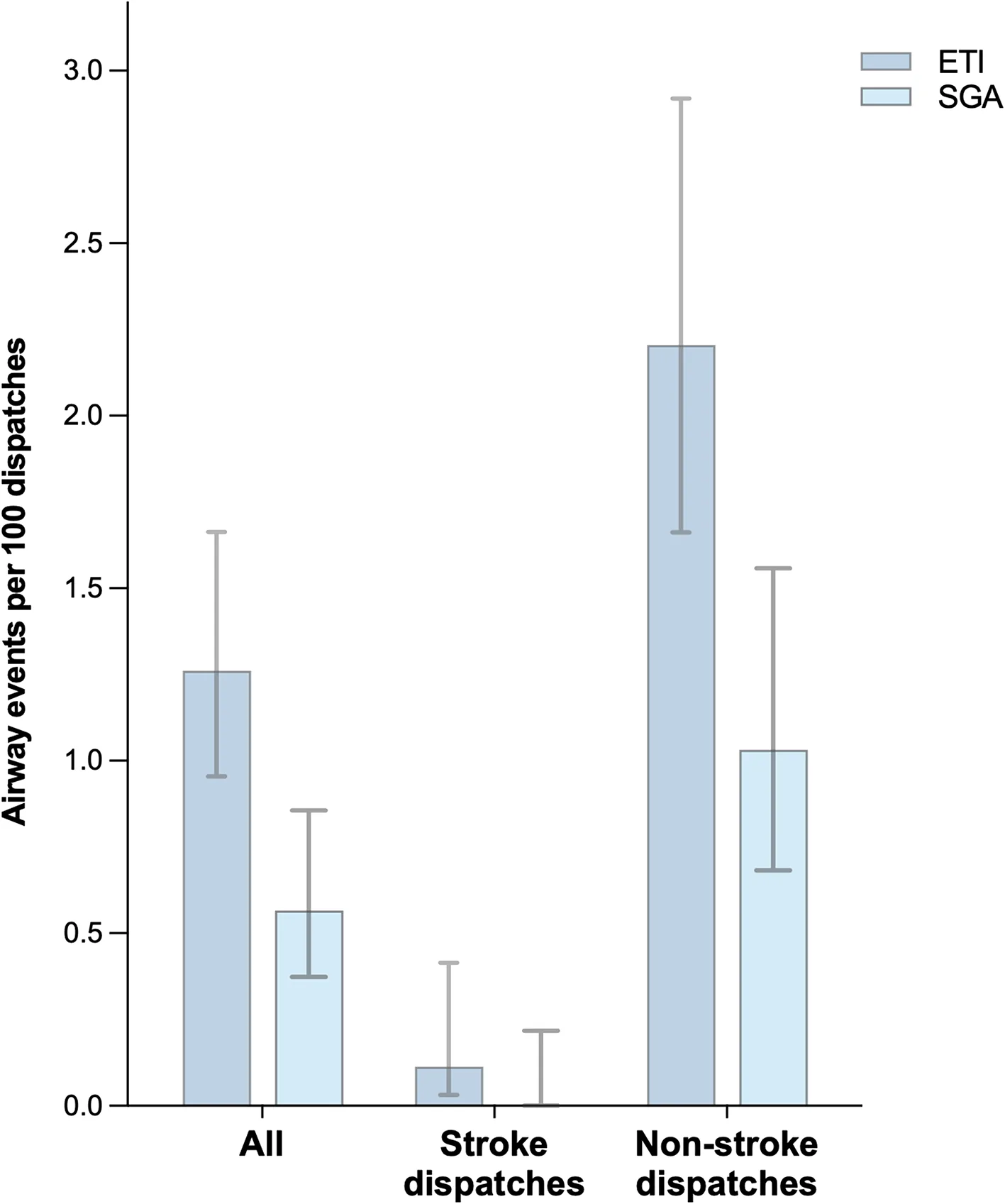
Dispatch-based ETI and SGA frequency Frequency of ETI and SGA use per 100 dispatches overall and by dispatch category. ETI occurred in 49/3,887 dispatches overall, including 2/1,755 stroke and 47/2,132 non-stroke dispatches. SGA use occurred in 22/3,887 dispatches all of which were non-stroke (22/2,132) dispatches. Error bars indicate 95% Wilson confidence intervals

ETI occurred in 49 of 3,887 dispatches (1.26%; 95% CI 0.95–1.66) and supraglottic airway use in 22 of 3,887 dispatches (0.57%; 95% CI 0.37–0.86). Airway interventions occurred mainly during non-stroke dispatches (Fig. 2). In stroke dispatches ETI was necessary in 2 of 1,755 dispatches (0.11%; 95% CI 0.03–0.41) with no use of supraglottic airway devices (0/1,755; 95% CI 0.00–0.22). In non-stroke dispatches ETI was performed in 47 of 2,132 dispatches (2.20%; 95% CI 1.66–2.92) and supraglottic airway use in 22 of 2,132 dispatches (1.03%; 95% CI 0.68–1.56). In seven cases of supraglottic airway use ETI was performed and the final airway device was an endotracheal tube, possibly indicating the sequential use of more than one airway device within the same mission. During the study period, six physicians performed the 49 ETIs recorded in this service. Individual case counts and dispatch-based frequencies for all nine participating physicians are presented in Supplementary Table 3. These data describe recorded activity within this service during the study period and do not represent total clinical intubation experience. Estimated previous prehospital intubation experience of the intubating physician was ≤ 100 intubations in 34 cases, 101–200 in 13 and > 200 in two. These estimates excluded intubations performed during clinical training and no not reflect overall clinical excperience. The Berlin emergency-physician training requirements are described in Supplementary Methods S1.

### Clinical indications for ETI

**Fig. 3:**
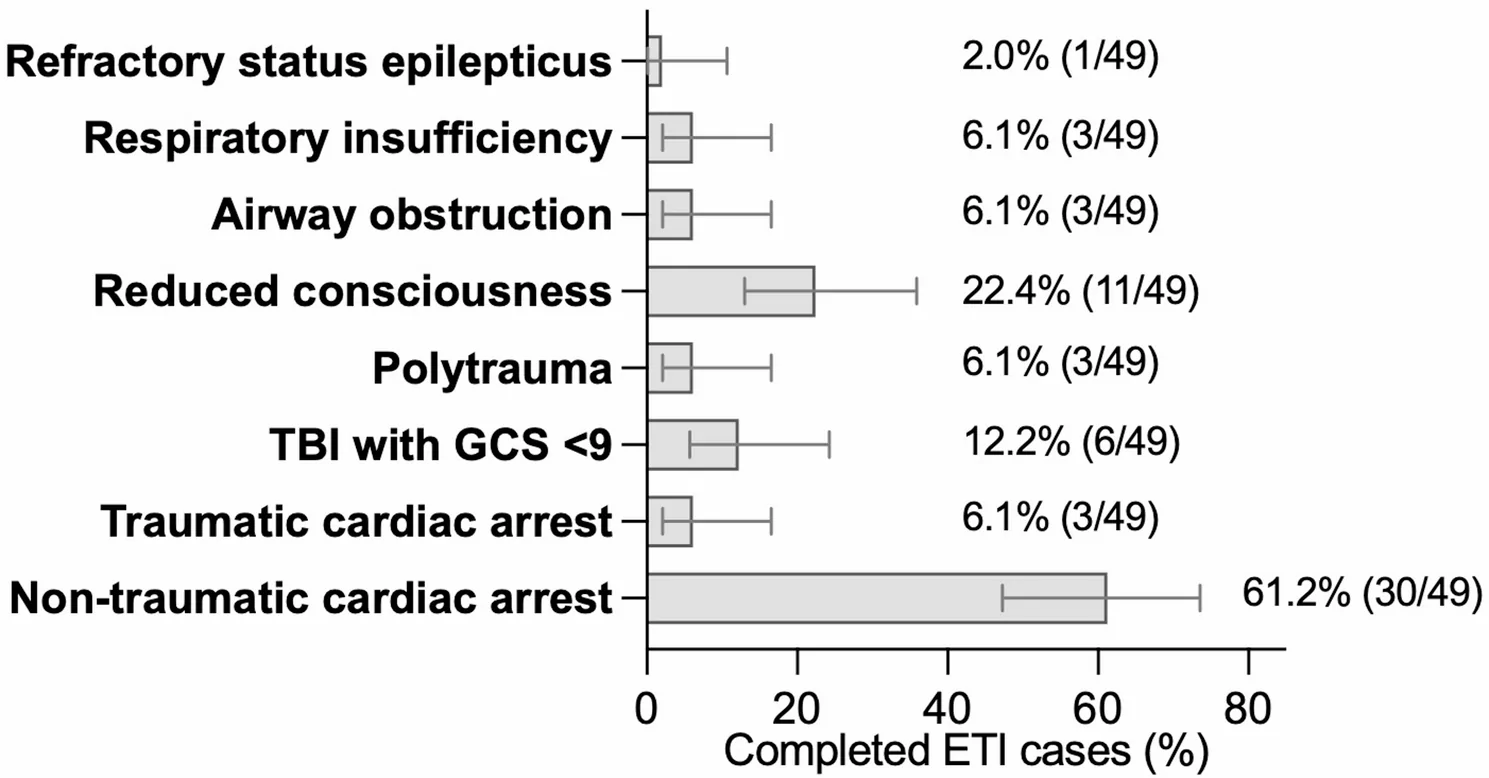
Clinical indications of ETI Clinical indications among completed ETI cases. Non-traumatic cardiac arrest was the most frequent indication (30/49; 61.2%). Multiple indications could be documented per case, so category counts may exceed the total number of ETIs. Error bars indicate 95% Wilson confidence intervals

Among 49 ETI cases the most frequent indication was non-traumatic cardiac arrest (30/49; 61.2%) (Fig. 3). Reduced consciousness (non traumatic brain injury related) was reported in 11/49 cases (22.4%), traumatic brain injury with Glasgow Coma Scale < 9 in 6/49 (12.2%) and traumatic cardiac arrest, polytrauma, airway obstruction and respiratory insufficiency in 3/49 cases each (6.1% each). In 1/49 ETI cases (2.0%) ETI was performed due to refractory status epilepticus. Because indications could be documented as multiple selections category counts could exceed the number of ETI cases. The documented two ETIs during stroke dispatches were performed for non-traumatic cardiac arrest and reduced consciousness secondary to intracerebral hemorrhage. Patient characteristics by dispatch category are presented in Supplementary Table 1. Age was recorded for 42/49 ETI cases, with a median of 66 years [IQR 58–81] and a range of 6–87 years. One patient was aged six years and seven ages were missing. Induction medication was recorded in 23/49 ETI cases (46.9%; Supplementary Table 2). Rocuronium was the only neuromuscular blocking agent used (*n* = 22). Among sedatives midazolam (*n* = 20) was recorded more frequently than propofol (*n* = 3). Among agents with analgesic properties esketamine (*n* = 13) was recorded more frequently than fentanyl (*n* = 8). All 26 cases without recorded induction medication had a cardiac arrest indication.

### First-pass success and glottic view

**Fig. 4:**
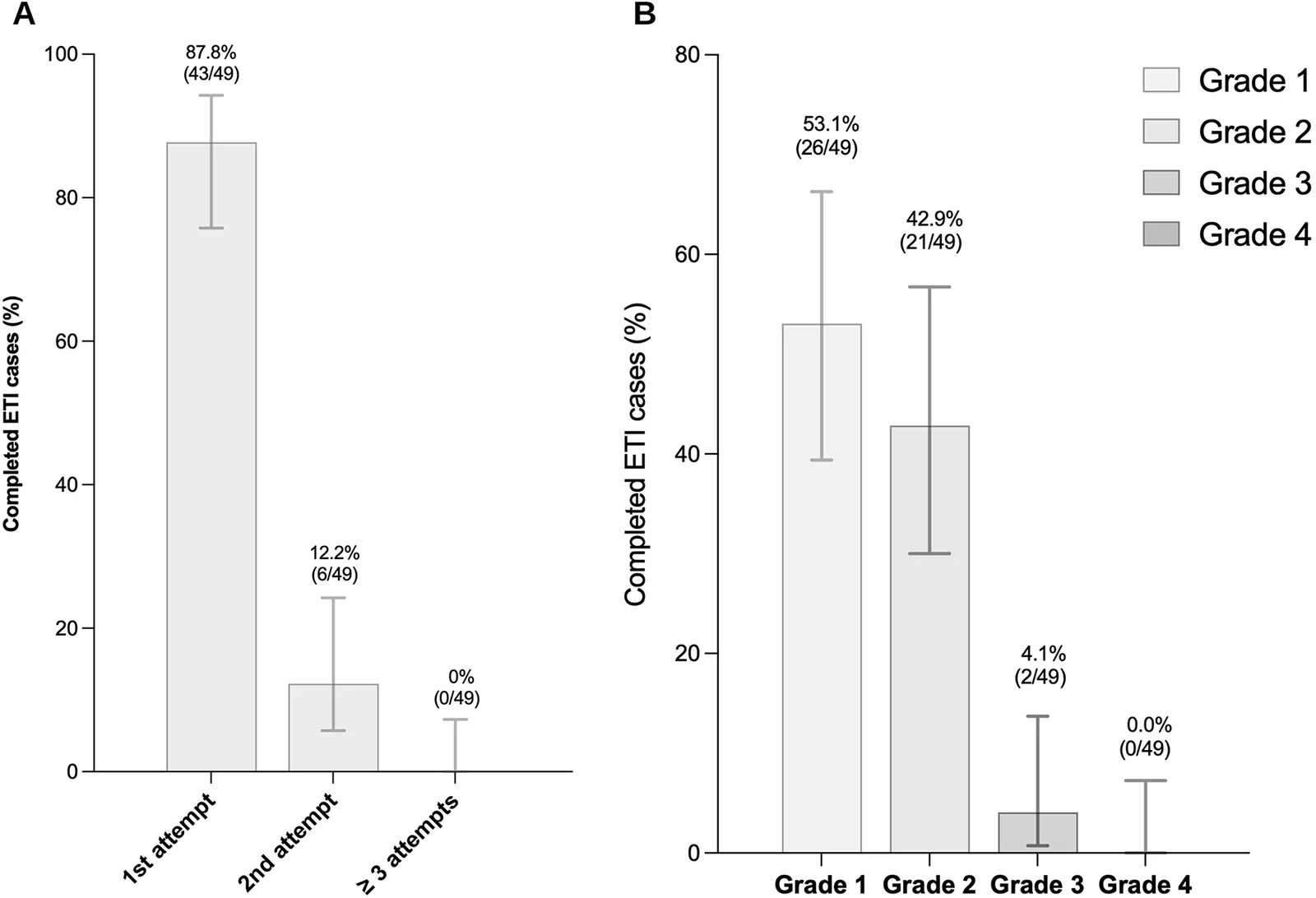
**A**. Number of ETI attempts. Distribution of intubation attempts among completed ETI cases. First-pass success was achieved in 43/49 cases (87.8%). Error bars indicate 95% Wilson confidence intervals. **B**. Glottic view. Distribution of Cormack-Lehane grades among completed ETI cases. Cormack-Lehane grades 1–2 were present in 47/49 cases (95.9%). Error bars indicate 95% Wilson confidence intervals

Overall intubation success was 49/49 (100.0%; 95% CI 92.7–100.0). First-pass success was achieved in 43/49 cases (87.8%; 95% CI 75.8–94.3) (Fig. 4A). Six of 49 ETIs (12.2%) were completed on the second attempt and no ETI case required more than two attempts. Cormack-Lehane grade was available in all ETI missions (Fig. 4B). Grade 1 was reported in 26/49 cases (53.1%), grade 2 in 21/49 (42.9%), grade 3 in 2/49 (4.1%) and grade 4 in 0/49. Cormack-Lehane grades 1–2 were accordingly present in 47/49 ETI cases.

### Difficult-airway indicators and procedural characteristics

**Fig. 5:**
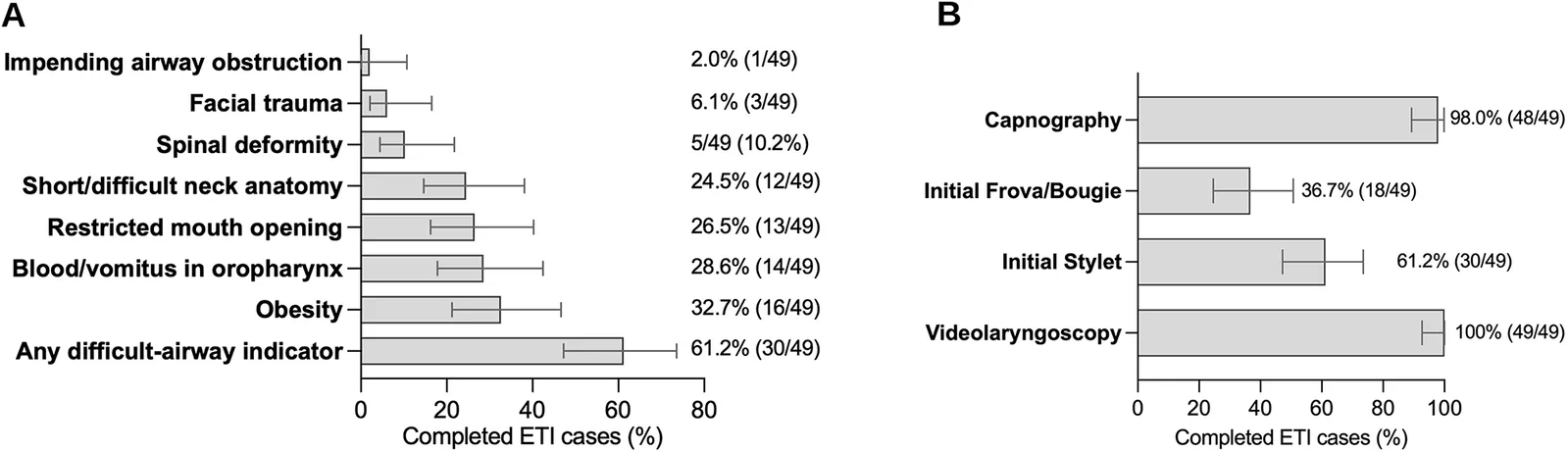
**A**. Difficult-airway indicators. Difficult-airway indicators among completed ETI cases. At least one indicator was present in 30/49 cases (61.2%). Multiple indicators could be documented per case, so category counts may exceed the total number of ETIs. Error bars indicate 95% Wilson confidence intervals. **B**. Airway technique and monitoring. Videolaryngoscopy was used in all ETI cases (49/49, 100.0%) and capnography in 48/49 cases (98.0%). Initial stylet or bougie was used in 48/49 ETI cases (97.96%). Error bars indicate 95% Wilson confidence intervals

At least one difficult-airway indicator was documented in 30/49 ETI cases (61.2%) (Fig. 5A). The most frequent indicators were obesity (16/49, 32.7%), blood or vomitus in the oropharynx (14/49, 28.6%), restricted mouth opening (13/49, 26.5%) and difficult neck anatomy (12/49, 24.5%). Spinal deformity was documented in 5/49 cases (10.2%), facial trauma in 3/49 (6.1%) and impending airway obstruction in 1/49 (2.0%). Difficult-airway indicators were recorded as multiple selections, resulting in a higher total number of documented indicators compared to the total number of ETI cases. No serious adverse airway-related events were reported. No emergency surgical airway was performed. Videolaryngoscopy was used in all 49 ETI cases (100.0%), reflecting the standardized airway approach of the Berlin EMS (Fig. 5B). An initial stylet was used in 30/49 cases (61.2%). A bougie was used initially in 18/49 cases (36.7%). Capnographic confirmation of tube position was documented in 48/49 ETI cases (98.0%). In the remaining case, auscultation was recorded as the confirmation method. The reason for not using capnography was not documented. Use of a standardised preparation protocol was recorded in 34/49 ETI cases (69.4%).

### NASA-TLX overall workload

**Fig. 6:**
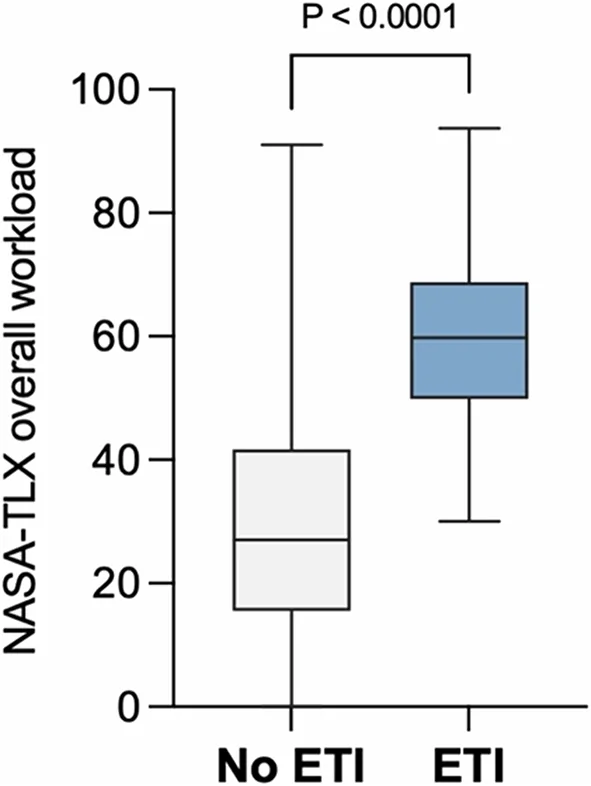
NASA-TLX overall workload. NASA-TLX overall workload in missions with and without ETI. Workload was higher in ETI missions than in missions without ETI (median 59.8 [IQR 50.0–68.7], n=49 vs 27.0 [IQR 15.5–41.7], n=2,700; Mann-Whitney U test, p<0.0001). Boxes indicate medians and interquartile ranges; whiskers indicate minimum to maximum values

NASA-TLX overall workload scores were available for 2,749 of 3,887 dispatches, including all 49 ETI cases. Missing scores were primarily due to missions being cancelled before arrival on scene. In the exploratory analysis overall self-reported workload was higher when ETI was performed than when it was not performed (median 59.8 [IQR 50.0–68.7] (*n* = 49) vs. 27.0 [IQR 15.5–41.7] (*n* = 2,700); Mann-Whitney U test, *p* < 0.0001, Fig. 6).

## Discussion

This retrospective registry analysis provides the first detailed real-world characterisation of advanced airway management in a neurologist-staffed MSU, including its frequency and the clinical indications in which it was required. MSUs are increasingly integrated into EMS worldwide, yet staffing models, dispatching criteria and non-stroke roles vary substantially between programmes [10, 17, 18, 27]. For example, in a Norwegian MSU model, anaesthesiologists with additional training in stroke assessment and cerebral CT interpretation served as the on-board physicians [28]. In the Berlin MSU service analysed here, neurologists with additional training in emergency medicine were deployed for both stroke and non-stroke dispatches. ETI and supraglottic airway use were uncommon at dispatch level. Airway interventions during stroke dispatches were especially rare, with only two ETIs and no SGA use in 1,755 stroke dispatches. Most airway exposure derived from deployments of the MSU as a general emergency physician vehicle, which lie outside the originally intended use case of MSUs. Although ETI was rarely required in this service, the on-board neurologists achieved a high first-pass success rate. Reported prehospital airway-management frequencies depend strongly on the case mix, operator model and attempt definition. In a Swiss physician-staffed EMS, ETI was attempted in 2,797 of 42,190 treated adult patients (6.6%) [29]. In the present service, ETI was performed in 49 of 3,887 dispatches (1.26%; 95% CI 0.95–1.66). We chose dispatches as the denominator because the comparison concerned stroke and non-stroke dispatch categories. The dispatch category is assigned at activation and before any information from the actual scene is available. Patient contact takes place later and its likelihood may differ between the groups. An analysis restricted to missions with patient contact would condition the comparison on a consequence of the exposure and could therefore introduce selection bias. Since the frequency of advanced airway management in this setting had not yet been described, we included every activation in each dispatch category. This measures the operational burden from the point at which the team is dispatched and health care resources are used. The frequencies we observed describe the current dispatch practice in the Berlin MSU service, including the use of the MSU as a general emergency physician vehicle. They should not be applied directly to MSUs dispatched for suspected stroke only or to systems with different EMS integration and dispatch criteria [10, 17, 18, 27]. The procedural results also cannot be assumed to apply to MSUs that rely on telemedicine support or on airway management by nurses or paramedics. ETI was performed mainly in high-acuity emergencies, particularly in non-traumatic cardiac arrest and these missions were associated with substantially higher self-reported workload. Routine MSU stroke dispatches apparently offer very limited opportunities to perform advanced airway management because ETI is very unfrequent. Maintaining hands-on exposure is therefore a particular concern for physician-staffed MSUs that attend suspected stroke only. Prehospital airway management is a high-risk procedure and requires trained clinicians, suitable equipment, established rescue strategies and appropriate clinical supervision [20, 30]. Trials and meta-analyses of prehospital airway management report relevant differences in intubation success between provider groups and EMS systems. Physician-staffed services and other highly trained teams often report higher success rates than less experienced groups [31–35]. The individual counts and dispatch-based frequencies in Supplementary Table 3 describe ETIs recorded within this service during the study period and do not include intubations performed during training, in hospital or in other services. Favourable intubation success rates were observed under the existing training and service conditions. This study was not designed to assess the adequacy of training or whether individual procedural exposure was sufficient to maintain competence. The training and refresher requirements of the Berlin EMS service are described in Supplementary Methods S1. Current UK guidance on prehospital anaesthesia emphasises assessment of competence and continued in-hospital anaesthesia practice [36]. Structured simulation and supervised clinical training may supplement limited prehospital exposure [20, 30, 37].

The high first-pass success rate observed in this registry analysis should be interpreted with caution. First-pass success is a clinically relevant measure of quality, because repeated laryngoscopy attempts are associated with more airway-related adverse events [38–40]. In this retrospective analysis, all 49 recorded ETIs were successful. First-pass success was achieved in 43 of 49 cases and all intubations were completed within two attempts. The German EMS system is physician-based. In Berlin, prehospital ETI is a physician responsibility and is usually performed by emergency physicians rather than paramedics, as reflected in local EMS protocols [41]. All ETIs in this study were performed by the on-board neurologist, who also held the additional qualification in emergency medicine required in Berlin. In the adult trauma cohort undergoing prehospital emergency anaesthesia reported by Price et al., first-pass success was 90.2% for physicians and 87.4% for critical care paramedics within mixed physician–paramedic teams [35]. For additional international context, in the paramedic-based AIRWAYS-2 trial of out-of-hospital cardiac arrest, successful initial ventilation with tracheal intubation within up to two attempts was reported in 1,891 of 2,723 patients (69.4%) allocated to and initially managed with tracheal intubation [42]. This endpoint differs from first-pass intubation success, and differences in case mix, provider roles and definitions limit direct comparison with our results. In particular, 30 of the 49 intubations were performed during non-traumatic cardiac arrest. Intubating conditions in these patients differ from those in the drug-assisted intubation cohorts, which are often reported in the literature [33–35, 43]. During the study period, the intubating physician’s estimated number of previous prehospital intubations was recorded for each ETI in three categories (≤ 100, 101–200 or > 200). Intubations performed during clinical training or hospital practice were not captured, so these estimates do not represent total clinical intubation experience. With only 49 ETIs, first-pass success cannot be reliably attributed to specialty, training or individual operators. The analysis nevertheless provides useful data on ETI performance in routine practice in this setting. More than half of the cases had difficult-airway indicators, yet the glottic view was favourable in 47 of 49 ETIs (Cormack–Lehane grade 1–2). The need for ETI in this MSU service was mainly related to high-acuity non-stroke emergencies. Non-traumatic cardiac arrest accounted for 30 of 49 ETIs and was the most common indication. For out-of-hospital cardiac arrest, provider competence and local practice remain major determinants of airway management. Large randomised trials have not identified a single airway strategy that is superior across all EMS systems. In the AIRWAYS-2 trial an initial supraglottic airway strategy was not superior to tracheal intubation for favourable functional outcome, whereas the PART trial reported higher 72-hour survival with an initial laryngeal-tube strategy compared with initial ETI. This retrospective analysis did not link airway device choice to patient outcomes during prehospital cardiac arrest [42, 44] and can therefore only describe local practice.

Videolaryngoscopy was documented in all ETI case and stylets or bougies were used in most cases. Similar device use is described in current prehospital and intensive care airway management literature. Systematic reviews of prehospital studies give mixed results for video versus direct laryngoscopy. Individual trials in physician-staffed prehospital services and in critically ill patients requiring emergency airway management suggest possible first-pass advantages with videolaryngoscopy [45–47]. Bougies were often used initially. This might reflect the predominance of high-acuity patients and difficult-airway indicators in the case mix of this study. A lower threshold for adjunct use among non-anaesthesiologist physicians might also contribute to this observation. Capnographic confirmation of tube position was used in 48 of 49 ETI cases, possibly reflecting current evidence-based practice, since continuous end-tidal carbon dioxide monitoring improves confirmation of correct tube placement and reduces the risk of unrecognised misplacement [20, 48].

NASA-TLX was developed to measure subjective workload [21]. Previous prehospital research has shown that workload varies with mission-level factors, including patient acuity and invasive procedures [49]. In our exploratory secondary analysis, overall NASA-TLX scores were substantially higher for missions involving ETI than for those without ETI. The association is clinically plausible, but does not specifically isolate the causal effect of intubation. ETI may instead mark the broader high-acuity context of a mission, including cardiac arrest, time pressure and simultaneous team tasks. Acute stroke care can also be demanding, but greater familiarity with stroke care than with infrequent airway procedures may influence perceived workload. Physicians from specialties with potentially less exposure to airway management during clinical training, including internal medicine, surgery and neurology, might perceive ETI as more demanding than anaesthesiologists, who routinely perform airway management as part of their clinical training. However, this study did not compare workload between specialties or assess its relationship with prior airway experience. The observed higher workload may also reflect the overall high-acuity context of these missions and cannot be attributed specifically to intubation.

## Conclusions

Advanced airway management was uncommon in this neurologist-staffed MSU service, particularly during stroke dispatches. Non-stroke dispatches accounted for most ETIs and non-traumatic cardiac arrest was the most common indication. Difficult-airway indicators were often recorded, but Cormack-Lehane grades were favourable and first-pass success was high. These findings describe the operational burden of airway management in a neurologist-staffed MSU under real-world dispatching practice, which currently also includes deployment to non-stroke emergencies beyond the intended stroke-specific use case. They support standardised prospective airway documentation and larger prospective studies comparing airway management across models of care.

## Supplementary Information

Below is the link to the electronic supplementary material.

Supplementary Material 1

## Data Availability

Data are available upon reasonable request.

## Abbreviations

AHA/ASA: American heart association/american stroke association
CI: Confidence interval
EMS: Emergency medical services
ETI: Endotracheal intubation
IQR: Interquartile range
MSU: Mobile stroke unit
NASA-TLX: National aeronautics and space administration task load index
PRESTO: Prehospital stroke treatment organization
REDCap: Research electronic data capture
SGA: Supraglottic airway
STEMO: Stroke emergency mobile
STROBE: Strengthening the reporting of observational studies in epidemiology
