# Supplementary material for: Airway management during missions of a neurologist-staffed mobile stroke unit: a retrospective registry study
Source: Scand J Trauma Resusc Emerg Med. 2026 Nov 9;34:174. doi: 10.1186/s13049-026-01726-x (PMC13753404; doi:10.1186/s13049-026-01726-x)
Supplement: Supplementary file 1 [file 13049_2026_1726_MOESM1_ESM.docx]

Supplementary material and methods

Supplementary Methods S1

Physician qualification and training

All physicians held the German additional qualification in prehospital emergency medicine (Zusatzbezeichnung Notfallmedizin). The formal Berlin emergency-physician qualification requirements apply irrespective of the physicians primary clinical specialty. The requirements below describe the Berlin qualification framework [1,2]:

- **Clinical training** The additional qualification in emergency medicine (WBO 2021) requires 24 months of inpatient training in a specialty with direct patient care, 6 months in intensive care and 6 months in anaesthesia or an emergency department (36 months in total). Rotations of at least 2 weeks each are required in paediatric anaesthesia or paediatrics, obstetrics, anaesthesia and an emergency department unless covered by a previous clinical rotation. Recognised courses can replace the paediatric, obstetric and anaesthesia placements [1,2].
- **Structured training and certification**
  - 80-hour course in general and specialised emergency care [1,2]
  - Berlin emergency physician course (Grundkurs Notarzt Berlin) and major incident course (Großschadenskurs 1) [3]
  - Courses in paediatric emergencies, emergency ultrasound and intensive care transport [3]
  - Instruction in radio communication and medical devices [3]
  - Valid certificates in advanced life support and prehospital trauma care [3]
  - Advanced airway course and paediatric life support recertification. In the study service, Advanced Cardiovascular Life Support (ACLS) and Pediatric Advanced Life Support (PALS) refresher training was required every two years and trauma refresher training every three years.
- **Prehospital missions** 100 supervised primary emergency missions in a physician-staffed ground vehicle or helicopter are required in the Berlin EMS service. At least 20 must be accompanied by an experienced emergency physician holding the prehospital emergency medicine qualification and up to 25 can be completed as simulation training [1,2].
- **Airway management** The qualifications cover capnography, emergency anaesthesia, airway management including difficult airways, bag-mask ventilation and the use of supraglottic airway devices. The target minimum number is 50 endotracheal intubations including videolaryngoscopy [2].

For each ETI recorded during the study period, the intubating physician reported an estimate of previous prehospital intubations in one of three categories: ≤100, 101–200 or >200. Intubations performed during prior clinical training or hospital practice were not captured. These estimates only describe previous prehospital experience at the time of documentation and not the number of intubations performed during the study period or total clinical experience.

| **Characteristic** | **All ETI n = 49** | **Stroke n = 2** | **Non-stroke n = 47** |
| --- | --- | --- | --- |
| Recorded ETI cases | 49 | 2 | 47 |
| Age, years, median [IQR] | 66 [58–81] (n=42) | 61 (n=1) | 68 [58–81] (n=41) |
| Age not recorded | 7 | 1 | 6 |
| GCS at indication, median [IQR] | 3 [3–3] (n=49) | 3 and 7 (n=2) | 3 [3–3] (n=47) |
| Non-traumatic cardiac arrest | 30/49 (61.2%) | 1/2 (50.0%) | 29/47 (61.7%) |
| Traumatic cardiac arrest | 3/49 (6.1%) | 0/2 (0.0%) | 3/47 (6.4%) |
| Traumatic brain injury with GCS <9 | 6/49 (12.2%) | 0/2 (0.0%) | 6/47 (12.8%) |
| Polytrauma | 3/49 (6.1%) | 0/2 (0.0%) | 3/47 (6.4%) |
| Reduced consciousness of other aetiology | 11/49 (22.4%) | 1/2 (50.0%) | 10/47 (21.3%) |
| Airway obstruction | 3/49 (6.1%) | 0/2 (0.0%) | 3/47 (6.4%) |
| Respiratory insufficiency | 3/49 (6.1%) | 0/2 (0.0%) | 3/47 (6.4%) |
| Refractory status epilepticus | 1/49 (2.0%) | 0/2 (0.0%) | 1/47 (2.1%) |
| Documented first-pass success | 43/49 (87.8%) | 2/2 (100.0%) | 41/47 (87.2%) |

Supplementary Table 1 Patient characteristics and intubation indications by dispatch category

Values are n/N (%) or median [interquartile range], unless otherwise stated. Age was available in 42 cases and ranged from 6 to 87 years. One patient was aged six years, 41 were adults and seven ages were missing. Multiple indications could be selected. ETI, endotracheal intubation; GCS, Glasgow Coma Scale; IQR, interquartile range.

| **Recorded induction medication** | **All ETI n = 49** | **Stroke n = 2** | **Non-stroke n = 47** |
| --- | --- | --- | --- |
| Recorded induction medication | 23/49 (46.9%) | 1/2 (50.0%) | 22/47 (46.8%) |
| Midazolam | 20/49 (40.8%) | 1/2 (50.0%) | 19/47 (40.4%) |
| Esketamine | 13/49 (26.5%) | 0/2 (0.0%) | 13/47 (27.7%) |
| Propofol | 3/49 (6.1%) | 0/2 (0.0%) | 3/47 (6.4%) |
| Fentanyl | 8/49 (16.3%) | 1/2 (50.0%) | 7/47 (14.9%) |
| Rocuronium | 22/49 (44.9%) | 1/2 (50.0%) | 21/47 (44.7%) |

Supplementary Table 2 Induction medication in endotracheal intubation cases

Values are n/N (%); percentages use all ETI cases in the respective dispatch category as the denominator. Multiple agents could be recorded for each case. ETI, endotracheal intubation.

| **Physician** | **Recorded dispatches** | **Recorded ETI cases** | **ETI cases per 100 dispatches** |
| --- | --- | --- | --- |
| P01 | 950 | 14 | 1.47 |
| P02 | 251 | 3 | 1.20 |
| P03 | 29 | 0 | 0.00 |
| P04 | 825 | 8 | 0.97 |
| P05 | 779 | 11 | 1.41 |
| P06 | 194 | 0 | 0.00 |
| P07 | 416 | 9 | 2.16 |
| P08 | 377 | 4 | 1.06 |
| P09 | 66 | 0 | 0.00 |
| Total | 3,887 | 49 | 1.26 |

Supplementary Table 3 Endotracheal intubations per physician during the study period

ETI frequency is expressed per 100 recorded dispatches. Frequencies describe activity within this registry and during the study period, not duty-hour exposure or patient-level intubation opportunities. Intubations performed in hospital or in other services were not captured. ETI, endotracheal intubation.

Supplementary references

1. Ärztekammer Berlin. Weiterbildungsordnung von 2004, 11. Nachtrag (konsolidierte Fassung). Abschnitt C, Nr. 29 Notfallmedizin, S. 150–152. In Kraft seit 27.07.2019. <https://www.aekb.de/fileadmin/migration/pdf/11-NT_konsolidierte_Fassung_WBO_final.pdf>

2. Ärztekammer Berlin. Weiterbildungsordnung vom 22.09.2021 einschließlich 1. Änderung vom 15.06.2022 und 2. Änderung vom 13.11.2024, in Kraft seit 01.04.2025. Abschnitt C, Nr. 33 Zusatz-Weiterbildung Notfallmedizin, S. 408–411. <https://www.aekb.de/fileadmin/01_aerzt-innen/weiterbildung/AEKB_Weiterbildungsordnung-von-2021-mit-Aenderung-2.pdf>

3. Breuer F, Dahmen J, Malysch T, Poloczek S. Strukturqualität im Berliner Notarztdienst: Funktionen, Qualifikationen und Kompetenzerhalt. Notfall Rettungsmed. 2023;26(8):602–610. https://doi.org/10.1007/s10049-022-01020-0
